# Mesoscale Steady-State Dynamics Modeling and Parametric Analysis of the Viscoelastic Response of Asphalt-Bonded Calcareous Sand

**DOI:** 10.3390/ma19061194

**Published:** 2026-03-18

**Authors:** Linyu Xie, Bowen Pang, Peng Cao, Jianru Wang, Zhifei Tan

**Affiliations:** 1 College of Architecture and Civil Engineering, Beijing University of Technology, Beijing 100124, China; xielinyu2023@126.com (L.X.); zhifeitan@bjut.edu.cn (Z.T.); 2 The Institute of Xi’an Aerospace Solid Propulsion Technology, Xi’an 710025, China; 13335397977@163.com; 3 Academy of Aerospace Solid Propulsion Technology, Xi’an 710025, China

**Keywords:** Asphalt-Bonded Calcareous Sand (ABCS), random aggregate model, dynamic modulus, steady-state dynamics, Interfacial Transition Zone (ITZ), size effect

## Abstract

Due to the complex mesostructure of calcareous sand, accurately predicting the mechanical response of Asphalt-Bonded Calcareous Sand (ABCS) is extremely challenging. This study pioneers the development of a mesoscale model for ABCS that explicitly incorporates the Interfacial Transition Zone (ITZ) via a random particle algorithm. To overcome the efficiency bottlenecks of traditional time-domain integration, this study establishes a mesoscale framework coupling a random polygonal aggregate algorithm with direct Steady-State Dynamics (SSD) analysis. A major advantage of this framework is its capacity for large-scale parametric sensitivity analysis; herein, 920 independent mesoscale models were generated and rapidly solved across the broadband frequency domain. The framework was rigorously validated, demonstrating high predictive accuracy for both the baseline calibration and an independent 12% asphalt content mixture (baseline R^2^ = 0.99, MAPE = 6.94%; independent validation R^2^ = 0.96, MAPE = 9.73%). Notably, the SSD approach completes calculations (10^−3^ to 10^3^ Hz) for 10 massive 300 mm RVEs in just 6.5 min. Leveraging this high-throughput capability, the extensive parametric analysis reveals that variations in maximum aggregate size negligibly impact the dynamic modulus under a constant volume fraction. Conversely, an optimal Interfacial Transition Zone (ITZ) thickness of ~75 µm was identified, representing a physical equilibrium between interfacial reinforcement and bulk binder cohesion. Furthermore, an analytical RVE size criterion of 1.7–5.3 times the maximum aggregate size is proposed to satisfy a 5% engineering error tolerance, providing a highly efficient numerical tool for the virtual mix design of reef pavements.

## 1. Introduction

Calcareous sand is a unique biogenic geomaterial widely distributed in coral reefs and coastal regions, including the South China Sea, the Red Sea, Australia, and the Persian Gulf [1,2,3]. In remote island and reef engineering projects, the scarcity of raw materials and prohibitive transportation costs present significant challenges. Therefore, the resource utilization of calcareous sand, based on the principle of in situ sourcing, has become a critical strategy for the sustainable development of offshore engineering. Currently, cement mortar products utilizing calcareous sand as a substitute for siliceous sand have been applied in construction projects in the South China Sea [4]. Furthermore, with the expanding scale of marine resource development, there are broad prospects for its application in embankment filling and airfield runway foundations [5,6].

However, deploying calcareous sand in flexible asphalt pavements presents unique material challenges. Distinct from terrigenous siliceous sand—which typically undergoes long-distance transport and physical weathering—calcareous sand is characterized by a highly developed intra-particle pore structure, a large specific surface area, and high crushability [7,8]. These intricate microstructural features induce complex inter-particle contact behaviors, resulting in pronounced internal friction and interlocking effects under load. Consequently, calcareous sand exhibits mechanical properties characterized by high shear stiffness and low damping ratios [8,9]. Despite this high shear stiffness, the highly porous nature inevitably leads to excessive asphalt binder absorption. This high asphalt consumption poses a significant economic constraint and performance challenge for Asphalt-Bonded Calcareous Sand (ABCS) pavements. Consequently, to balance economic feasibility with engineering performance, it is critical to precisely evaluate and optimize mixture design parameters (such as binder content and interfacial properties) based on fundamental mechanical mechanisms. Therefore, while utilizing in situ calcareous sand is logistically necessary, its feasibility as an aggregate substitute requires in-depth mesoscale exploration.

To enhance the engineering applicability of calcareous sand, existing studies have predominantly focused on stabilization and modification [10,11,12,13,14,15,16]. Nevertheless, to mitigate the risks of uneven settlement inherent to island reef environments and to meet the serviceability requirements of high-grade transportation infrastructure such as runways, the construction of flexible asphalt pavements is essential [17]. Unlike static loading scenarios, pavement structures endure long-term reciprocating dynamic loads from vehicles. Under these conditions, the Dynamic Modulus ($\left|E^{*}\right|$) replaces the conventional static modulus as the core indicator characterizing the material’s viscoelastic response [18,19], directly determining rutting and fatigue resistance [20,21]. Currently, dynamic modulus acquisition relies on laboratory testing, empirical (or semi-empirical) models, and micromechanical methods. While laboratory results [22,23] are reliable, conducting high-precision dynamic tests on remote islands faces logistical challenges regarding equipment transport and extended sample preparation cycles. Empirical models, such as the Witczak [24,25], Hirsch [26,27], and Artificial Neural Network (ANN) models [28,29,30], typically offer high prediction accuracy only within their specific calibration ranges; their applicability is uncertain when directly extrapolated to novel calcareous sand systems. Furthermore, neither macroscopic experiments nor empirical models can fully elucidate the complex interaction mechanisms between components within ABCS. Therefore, it is necessary to investigate the internal mechanical feedback mechanisms at the mesoscale.

ABCS is fundamentally a highly heterogeneous multiphase composite, where the macroscopic viscoelastic response originates from complex mesoscale interactions [31,32]. Physical and chemical interactions between the asphalt mastic matrix and aggregates create a narrow Interfacial Transition Zone (ITZ) around aggregate particles [33]. Existing two-dimensional (2D) mesoscale studies confirm that explicitly incorporating the ITZ not only reflects the material structure more realistically but also demonstrates that ITZ thickness and properties significantly control low-temperature performance and viscoelastic behavior [34,35]. Moreover, local mechanical properties in the minute contact zones between aggregates determine the complex modulus of the asphalt mixture and are a primary factor in the tension–compression asymmetry of its macroscopic response [36,37]. For calcareous sand systems, the interfacial and mesoscale mechanisms are particularly complex. The developed intra-particle pores of calcareous sand possess strong physical adsorption capabilities [38,39,40], potentially causing the asphalt binder to migrate into the particles, thereby altering the effective thickness and physical modulus of the ITZ. Additionally, the angularity of coarse aggregates is key to skeletal strength, while particle size distribution directly modifies internal stress chain transmission paths, modulating the macroscopic modulus [41,42]. However, existing research on calcareous sand-bonded materials has largely focused on static or quasi-static properties, such as elastic modulus and shear behavior [43,44,45,46,47,48], or strictly on the improvement of asphalt mastic rheology when using calcareous sand as a filler [49]. There is currently a lack of systematic understanding regarding how key mesostructural parameters, such as ITZ thickness, affect the broadband dynamic response of calcareous sand systems, which severely restricts the promotion of this material in high-performance pavement structures.

In the realm of mesoscale asphalt mixture modeling, existing studies have successfully utilized image-based virtual modeling and multiscale micromechanical approaches to explicitly simulate multiphase structures and capture localized mechanical interactions—such as complex aggregate contact behaviors and ITZ effects [34,35,36,37]. However, analyzing these cross-scale mechanisms presents significant computational challenges. A critical limitation remains regarding broadband computational efficiency. Analytical micromechanical models based on equivalent inclusion theory [50,51,52,53,54] often rely on idealized particle assumptions and fail to capture skeletal reinforcement. In numerical simulations, pre-processing algorithms are often inefficient when handling complex heterogeneous models containing ITZs. The literature indicates that generating just four asphalt concrete finite element models with refined ITZs can take approximately 19 h [42], a bottleneck that severely hinders large-scale parameter sensitivity analysis. Furthermore, in the dynamic response solution phase, while both the Discrete Element Method (DEM) [55,56,57,58,59] and traditional Finite Element Method (FEM) [60] can solve for dynamic modulus, they primarily rely on transient time-domain step-by-step integration (e.g., the central difference or Newmark methods), which requires extremely small time increments to ensure convergence. Constructing a full-frequency dynamic modulus master curve using these time-domain solvers demands exorbitant computational time, severely hindering large-scale parametric sensitivity analyses. In contrast, the Steady-State Dynamics (SSD) method, based on linear perturbation theory, obtains the dynamic modulus by solving the stiffness matrix once, significantly improving computational efficiency while maintaining accuracy [61,62,63].

Addressing these critical gaps, and leveraging the superior efficiency of the direct SSD algorithm, this study establishes a high-performance numerical simulation framework. To the best of our knowledge, this study pioneers the application of a direct SSD framework coupled with a random polygonal aggregate generation algorithm to explicitly model the multiphase mesostructure of ABCS. It must be emphasized that the distinct scientific novelty and methodological advancement of this research extend well beyond the mere application of existing SSD algorithms or the simple integration of standard modeling techniques. Instead, this work creates a highly efficient, high-throughput computational pipeline specifically tailored for the broadband viscoelastic prediction of highly porous systems. By formulating and solving the complex stiffness matrix directly in the frequency domain, this framework completely bypasses the prohibitive time-domain integration bottlenecks characteristic of conventional FEM. This distinctive computational advantage enables the rapid generation and solution of an unprecedented 920 independent mesoscale models across a broad frequency spectrum (10^−3^ to 10^3^ Hz). Leveraging this massive parametric sweep capability, this study systematically decouples and quantifies the influence of maximum aggregate size, ITZ physical properties, asphalt binder content, and Representative Volume Element (RVE) size on the macroscopic broadband dynamic modulus. The results not only deepen the understanding of the mesoscale mechanisms controlling the viscoelastic behavior of ABCS but also provide quantitative RVE criteria and a highly efficient numerical tool for the virtual mix design of this eco-friendly pavement material.

## 2. Theoretical Methodology

### 2.1. Viscoelastic Theory

Under normal service conditions, ABCS is typically characterized as a viscoelastic material. To accurately describe and predict its stress-strain relationship under varying operating conditions, this section systematically presents the fundamental framework of linear viscoelastic theory.

The foundation of linear viscoelasticity is the Boltzmann superposition principle, which utilizes the strain step function: $\varepsilon (t)=\varepsilon_{0}H(t)$. In this equation, *H*(*t*) represents the Heaviside step function, defined as follows:

(1) $$H(t)=\left\{\begin{array}{cc} 0, & \mathrm{if}\;t<0,\\ 1/2, & \mathrm{if}\;t=0,\\ 1, & \mathrm{if}\;t>0. \end{array}\right.$$

To predict the stress response for an arbitrary strain history, rather than a single strain step, we decompose the total strain history into a summation of infinitesimal strain steps [64]:

(2) $$\varepsilon (t)=\sum_{i=1}^{\infty }\Delta \varepsilon_{i}H(t-\tau_{i}),$$

where $\Delta \varepsilon_{i}$ represents the strain increment applied at time $\tau_{i}$. Consequently, the total stress response to this strain history can be obtained via the superposition principle:

(3) $$\sigma (t)=\sum_{i=1}^{\infty }\Delta \varepsilon_{i}E_{R}(t-\tau_{i}).$$

here, $E_{R}(t)$ denotes the stress relaxation modulus, defined as $E_{R}(t)\equiv \sigma (t)/\varepsilon_{0}$. As the number of strain increments approaches infinity, the stress response can be expressed in integral form [65]:

(4) $$\sigma (t)=\int_{-\infty }^{t}E_{R}(t-\tau )d\varepsilon (t)=\int_{-\infty }^{t}E_{R}(t-\tau )\frac{d\varepsilon (\tau )}{d\tau }d\tau$$

once $E_{R}(t)$ is determined, the integral equation can be employed to predict the stress response resulting from any applied strain history. Typically, the relaxation modulus is characterized using the Generalized Maxwell model in the form of a Prony series:

(5) $$E_{R}(t)=E_{e}+\sum_{i=1}^{N}E_{i}e^{-t/\tau_{i}}$$

where $E_{e}$ is the equilibrium modulus, while $E_{i}$ and $\tau_{i}$ represent the spring modulus and relaxation time of the *i*-th Maxwell element, respectively.

### 2.2. Dynamic Modulus

Under dynamic loading, the mechanical response of ABCS exhibits both time and frequency dependence. When subjected to a sinusoidal strain, the resulting steady-state stress response, σ(t) is also sinusoidal.

Consider a strain history defined by:

(6) $$\varepsilon (t)=\left\{\begin{array}{cc} \varepsilon_{0}\sin(\omega t), & \mathrm{if}\;t\geq 0,\\ 0, & \mathrm{if}\;t<0. \end{array}\right.$$

The stress response to this strain history can be derived from Equation (4) as follows:

(7) $$\sigma (t)=\int_{0}^{\infty }E(s)\omega \varepsilon_{0}\cos\left[\omega (t-s)\right]ds$$

where s≡t−τ. Equation (7) can be expanded as follows:

(8) $$\sigma (t)=\varepsilon_{0}\sin(\omega t)\left[\omega \int_{0}^{\infty }E(s)\sin(\omega s)ds\right]+\varepsilon_{0}\cos(\omega t)\left[\omega \int_{0}^{\infty }E(s)\cos(\omega s)ds\right]$$

By defining the storage modulus, $E^{\prime }(\omega )$, and the loss modulus, $E^{\prime \prime }(\omega )$, Equation (8) can be rewritten as follows [64]:

(9) $$\sigma (t)=\varepsilon_{0}\left[E^{\prime }(\omega )\sin(\omega t)+E^{\prime \prime }(\omega )\cos(\omega t)\right]$$

where, $E^{\prime }(\omega )=\omega \int_{0}^{\infty }E(s)\sin(\omega s)ds,\;E^{\prime \prime }(\omega )=\omega \int_{0}^{\infty }E(s)\cos(\omega s)ds$.

Alternatively, the stress response can be expressed as follows:

(10) $$\sigma (t)=\sigma_{0}\sin(\omega t+\delta )=\sigma_{0}\sin(\omega t)\cos\delta +\sigma_{0}\cos(\omega t)\sin\delta$$

Comparing the coefficients of the sine and cosine terms in Equations (9) and (10) yields the following:

(11) $$\varepsilon_{0}E^{\prime }(\omega )=\sigma_{0}\cos\delta$$

(12) $$\varepsilon_{0}E^{\prime \prime }(\omega )=\sigma_{0}\sin\delta$$

From which the phase angle, δ is obtained:

(13) $$\tan\delta =\frac{E^{\prime \prime }}{E^{\prime }}$$

The complex modulus, $E^{*}$, is then introduced using complex number notation:

(14) $$E^{*}=\frac{\sigma^{*}}{\varepsilon^{*}}=E^{\prime }+iE^{\prime \prime }=\left|E^{*}\right|(\cos\delta +i\cdot \sin\delta )$$

where $|E^{*}|=\sigma_{0}/\varepsilon_{0}$ represents the dynamic modulus, and $\delta =\arctan(E^{\prime \prime }/E^{\prime })$ is the phase angle.

In pavement engineering, asphalt mixtures show strong temperature- and frequency-dependent behavior. To account for temperature effects and characterize mechanical behavior across a broad frequency range, we employ the Time–Temperature Superposition Principle (TTSP) to construct a master curve. This principle calculates a shift factor, $a_{T}$, to horizontally translate dynamic modulus data from actual physical test temperatures into a unified “reduced frequency” domain at a designated reference temperature, $T_{0}$. The shift factor is defined as follows:

(15) $$\log t_{T_{0}}-\log t_{T}=\log a_{T}$$

where $t_{T}$ is the time at the test temperature *T*, and $t_{T_{0}}$ is the corresponding time at the reference temperature $T_{0}$.

The temperature-dependent shift factor is typically described by the Williams-Landel-Ferry (WLF) equation [66]:

(16) $$\log a_{T}(T)=\frac{C_{1}(T-T_{0})}{C_{2}+T-T_{0}}$$

where $C_{1}$ and $C_{2}$ are empirical material constants.

In this study, the 2S2P1D model, widely used in asphalt rheology, is selected as the theoretical benchmark. The analytical expression for the complex dynamic modulus in the frequency domain is given by the following [67]:

(17) $$E^{*}=E_{0}+\frac{E_{g}-E_{0}}{1+\alpha {\left(i\omega \tau \right)}^{-k}+{\left(i\omega \tau \right)}^{-h}+{\left(i\omega \beta \tau \right)}^{-1}}$$

In this equation, *k* and *h* are exponents such that 0 < *k* < *h* < 1; α is a constant; $E_{0}$ is the static (equilibrium) modulus as ω→0; $E_{g}$ is the glassy modulus as ω→1; and β is a constant defined by the following:

(18) $$\beta =\frac{\eta }{(E_{g}-E_{0})\tau }$$

where η is the Newtonian viscosity and τ is the characteristic relaxation time.

### 2.3. Random Aggregate Modeling Method

To accurately predict the global viscoelastic behavior and mesoscale response of ABCS, the material is conceptualized at the mesoscale as a three-phase composite consisting of calcareous aggregates, the ITZ, and the asphalt mastic matrix. Currently, computational micromechanics methods for constructing asphalt mixture mesostructures fall into two primary categories. The first, Digital Image Processing (DIP), reconstructs mesoscopic geometry by analyzing real specimen images obtained via X-ray Computed Tomography (CT) or digital photography. However, this method is limited by its reliance on physical specimens, making sample preparation time-consuming and image acquisition costly. The second category employs random aggregate generation algorithms. These methods generate aggregate morphologies based on specific algorithms without requiring physical specimens, offering superior efficiency in time and cost, as well as broader applicability. Various generation algorithms have been developed to date [68,69,70,71,72].

To precisely reproduce the heterogeneous mesostructure of ABCS, this study adopts and optimizes a multi-step stochastic algorithm based on the Monte Carlo method to generate two-dimensional RVEs that are statistically representative and geometrically realistic. The algorithm ensures model reliability through a series of rigorous geometric and statistical constraints. The core procedure comprises the following key steps:

(1) Generation of Random Polygonal Aggregates

First, the number of edges for a polygon, *N_edge_*, is randomly selected from a uniform distribution within a preset range [*N_min*, *N_max*]. Subsequently, based on a polar coordinate system, random perturbations are applied to the radial and angular coordinates of each vertex *i* (*i* = 0, 1, …, *N_edge_*−1) to simulate the morphological characteristics of natural aggregates:

(19) $$r_{i}=r\left(1+\alpha_{r}\cdot U\left[-1,1\right]\right)$$

(20) $$\theta_{i}=\frac{2\pi }{N_{edge}}\left(i+\alpha_{\theta }\cdot U\left[-1,1\right]\right)$$

where r is the reference radius; $\alpha_{r}$ and $\alpha_{\theta }$ are dimensionless parameters controlling the degree of randomization for the radius and angle, respectively; and $U\left[-1,1\right]$ represents a random number uniformly distributed over the interval $\left[-1,1\right]$.

(2) Shape Quality Control

To prevent the generation of aggregates with extreme geometries (e.g., excessively elongated or concave shapes)—which could induce mesh generation difficulties and computational distortion—a shape factor, S, is introduced for morphological quality control:

(21) $$S=\frac{P^{2}}{4\pi A}$$

where P and A denote the perimeter and area of the aggregate, respectively. An aggregate is accepted only if S is less than a preset threshold, $S_{max}$; otherwise, it is regenerated until the morphological constraints are satisfied.

(3) Monte Carlo Aggregate Placement Based on Multiple Constraints

The spatial distribution of aggregates within the RVE is achieved using Monte Carlo Rejection Sampling. For each aggregate to be placed, the algorithm performs a multi-constraint check on its candidate position:

- Boundary Constraint: Ensures the entire aggregate (including its potential interface layer) lies completely within the predefined boundaries of the RVE.
- Overlap Constraint: To strictly prevent physical overlap between particles, a two-stage detection method is employed. First, a rapid bounding circle check is performed. If the circumscribed circles of two aggregates do not intersect, they are deemed non-overlapping. If they do intersect, the more precise Ray Casting Algorithm is triggered. This determines overlap by calculating the parity of intersection points between a ray emanating from within the candidate aggregate and the boundaries of existing aggregates.
- Minimum Spacing Constraint: To explicitly account for the ITZ in the model, the algorithm enforces a minimum distance between any two aggregates that is no less than the preset ITZ thickness.

A placement is accepted only when a candidate position simultaneously satisfies all the above constraints. If a suitable position is not found within a preset maximum number of attempts (e.g., 2000 iterations), the aggregate placement is declared a failure to avoid infinite loops.

(4) Gradation-Based Statistical Control and Optimized Placement Strategy

To ensure the generated RVE statistically conforms to the macroscopic material gradation, the algorithm strictly follows a preset aggregate gradation table. The system calculates the number of aggregates required for each particle size range based on target area percentages. The placement process adopts a “large-to-small” strategy, prioritizing the placement of larger aggregates. Practice has shown that this strategy significantly improves the success rate of placement and the final packing density. It reserves space for large aggregates while allowing smaller aggregates to fill the interstitial voids, better reflecting natural packing processes.

(5) Multi-Phase Identification and Material Property Assignment

**Figure 1:**
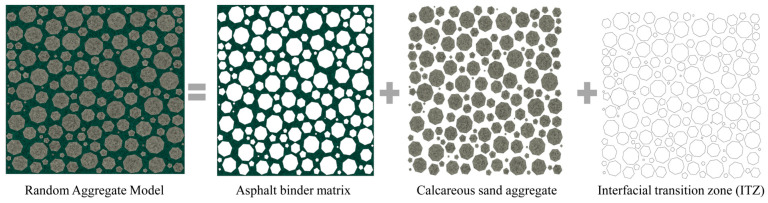
Multiphase mesoscale finite element model of ABCS.

Upon the completion of aggregate placement, the RVE is discretized into finite elements. The algorithm traverses all elements in the mesh and, by calculating the positional relationship of each element’s centroid relative to the aggregate boundaries, automatically identifies and classifies each element into one of three phases: aggregate, ITZ, or asphalt binder matrix. Finally, the system assigns the corresponding constitutive models and mechanical parameters to the element sets of each phase, forming a multi-phase mesoscale model ready for direct finite element analysis, as shown in Figure 1.

In summary, by combining parametric geometric generation, multi-constraint random placement, and gradation-based statistical control, this algorithm efficiently constructs numerical models that satisfy both macroscopic statistical characteristics and realistic mesostructural requirements. This provides a solid foundation for the subsequent prediction of mesoscale mechanical performance. However, while the current 2D model incorporates a distinct ITZ phase, it omits the explicit geometric representation of intra-aggregate porosity and localized binder absorption. Instead, these complex microstructural features are homogenized into the effective moduli of the aggregate and ITZ phases—a modeling simplification that will be addressed in future multi-phase studies.

### 2.4. Steady-State Dynamics Numerical Method

Predicting the viscoelastic response of ABCS across a broadband frequency domain using traditional time-domain stepwise integration methods presents severe computational efficiency bottlenecks. To address this, this study employs the direct SSD method as the core solution strategy. Based on linear perturbation theory, this method transforms the time-dependent dynamic problem into a system of complex linear equations in the frequency domain. This approach allows for the direct acquisition of the structure’s dynamic modulus without the need for cumbersome time-domain iterations [61,63]. This approach significantly enhances computational efficiency. Moreover, it provides a high-precision numerical pathway for constructing full-frequency dynamic modulus master curves consistent with the TTSP.

The direct steady-state dynamics method is founded on the dynamic virtual work equation [73]:

(22) $$\int_{V}\rho \delta u\cdot \ddot{u}dV+\int_{V}\rho \alpha_{c}\delta u\cdot \dot{u}dV+\int_{V}\delta \varepsilon :\sigma dV-\int_{S_{t}}\delta u\cdot tdS=0$$

where $\dot{u}$ and *ü* denote velocity and acceleration, respectively; ρ is the material density; $\alpha_{c}$ is the mass-proportional damping coefficient; σ represents stress; and δε is the strain variation consistent with the virtual displacement δu.

The discretized form of Equation (22) is expressed as follows:

(23) $$\delta u^{N}\left\{M^{NM}{\ddot{u}}^{M}+C_{\left(m\right)}^{NM}{\dot{u}}^{M}+I^{N}-P^{N}\right\}=0$$

where:

(24) $$\left\{\begin{array}{l} M^{NM}=\int_{V}\rho N^{N}\cdot N^{M}dV\\ C_{\left(m\right)}^{NM}=\int_{V}\rho \alpha_{c}N^{N}\cdot N^{M}dV+\alpha_{g}M^{NM}\\ I^{N}=\int_{V}\beta^{N}:\sigma dV\\ P^{N}=\int_{S_{t}}N^{N}\cdot tdS \end{array}\right.$$

in these equations, $M^{NM}$ is the mass matrix; $C_{\left(m\right)}^{NM}$ is the mass-proportional damping matrix; $I^{N}$ is the internal load vector; $P^{N}$ is the external load vector; $N^{N}$ is the shape function matrix; $\beta^{N}$ is the strain-displacement matrix; and $\alpha_{g}$ is the damping parameter.

For steady-state harmonic response, the structure is assumed to undergo small harmonic oscillations around a base deformed and stressed state (denoted by the subscript 0). Since steady-state dynamics is a linear perturbation procedure, the loads and responses in this step define variations relative to the base state. The variation of the internal force vector is obtained via linearization:

(25) $$\Delta I^{N}=\int_{V}\left[\Delta \beta^{N}:\sigma +\beta^{N}:\Delta \sigma \right]dV$$

The stress variation can be written as follows:

(26) $$\Delta \sigma =D^{el}:(\Delta \varepsilon +is\Delta \varepsilon +\beta_{c}\Delta \dot{\varepsilon })$$

where $D^{el}$ is the elasticity matrix of the material; $\beta_{c}$ is the stiffness-proportional damping coefficient (part of the Rayleigh damping assumption); and *s* is the structural damping coefficient, which constitutes the imaginary part of the stiffness matrix (i.e., the structural damping matrix). It should be noted that the input of Rayleigh damping coefficients is fundamentally not required in our model. Within the direct steady-state dynamics (SSD) framework, the energy dissipation of the system is intrinsically and entirely captured by the complex modulus of the frequency-dependent viscoelastic constitutive model. The variations in strain and strain rate are derived from the variations in displacement and velocity:

(27) $$\Delta \varepsilon =\beta^{M}\Delta u^{M},\;\Delta \dot{\varepsilon }=\beta^{M}\Delta {\dot{u}}^{M}$$

Consequently, Equation (23) can be reformulated as follows:

(28) $$\delta u^{N}\left\{M^{NM}{\ddot{u}}^{M}+(C_{\left(m\right)}^{NM}+C_{\left(k\right)}^{NM}){\dot{u}}^{M}+(iC_{\left(s\right)}^{NM}+K^{NM})u^{M}-P^{N}\right\}=0$$

The stiffness matrix $K^{NM}$, stiffness-proportional viscous damping matrix $C_{\left(k\right)}^{NM}$, and stiffness-proportional structural damping matrix $C_{\left(s\right)}^{NM}$ are defined as follows:

(29) $$\left\{\begin{array}{l} K^{NM}=\int_{V}\left[\frac{\partial \beta^{N}}{\partial u^{M}}:\sigma_{0}+\beta^{N}:D^{el}:\beta^{M}\right]dV\\ C_{\left(k\right)}^{NM}=\int_{V}\beta_{c}\beta^{N}:D^{el}:\beta^{M}dV+\beta_{g}K^{NM}\\ C_{\left(s\right)}^{NM}=\int_{V}s\beta^{N}:D^{el}:\beta^{M}dV+s_{g}K^{NM} \end{array}\right.$$

For harmonic excitation and response:

(30) $$\Delta u^{M}=\left(ℜ\left(u^{M}\right)+iℑ\left(u^{M}\right)\right)\exp i\Omega t$$

(31) $$\Delta P^{N}=\left(ℜ\left(P^{N}\right)+iℑ\left(P^{N}\right)\right)\exp i\Omega t$$

where $ℜ\left(u^{M}\right)$ and $ℑ\left(u^{M}\right)$ are the real and imaginary parts of the displacement amplitude; $ℜ\left(P^{N}\right)$ and $ℑ\left(P^{N}\right)$ are the real and imaginary parts of the force amplitude applied to the structure; and Ω is the circular frequency.

Substituting Equations (30) and (31) into Equation (28) yields the complex linear system:

(32) $$\left.\left[\begin{array}{cc} ℜ\left[A^{NM}\right] & ℑ\left[A^{NM}\right]\\ ℑ\left[A^{NM}\right] & -ℜ\left[A^{NM}\right] \end{array}\right.\right]\left\{\begin{array}{c} ℜ\left(u^{M}\right)\\ ℑ\left(u^{M}\right) \end{array}\right\}=\left\{\begin{array}{c} ℜ\left(P^{N}\right)\\ -ℑ\left(P^{N}\right) \end{array}\right\}$$

where the complex system matrix $A^{NM}$ is given by the following:

(33) $$\left\{\begin{array}{l} ℜ\left[A^{NM}\right]=K^{NM}-\Omega^{2}M^{NM}\\ ℑ\left[A^{NM}\right]=-\Omega (C_{\left(m\right)}^{NM}+C_{\left(k\right)}^{NM})-C_{\left(s\right)}^{NM} \end{array}\right.$$

This procedure is activated in ABAQUS 2023 (Dassault Systèmes Simulia Corp., Johnston, RI, USA) by defining a direct-solution steady-state dynamic analysis step. In this analysis, the steady-state harmonic response is calculated directly using the system’s mass, damping, and stiffness matrices based on the model’s physical degrees of freedom. The output provides the amplitude and phase of all element and nodal variables at the requested frequencies.

**Figure 2:**
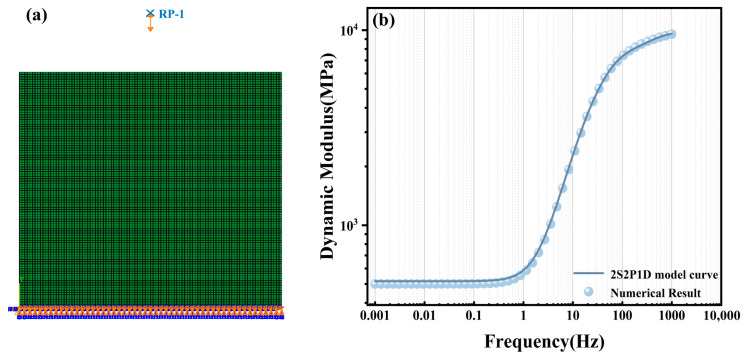
Comparison between the 2S2P1D analytical solution and the SSD numerical solution: (**a**) Homogeneous SSD numerical model. (**b**) Comparison of analytical and numerical solutions.

To verify the accuracy of the steady-state dynamics module in simulating complex viscoelastic behavior, a validation study was conducted comparing numerical solutions obtained via SSD against analytical solutions derived from the 2S2P1D theoretical model. Table 1 lists the parameters adopted for the 2S2P1D model, from which the analytical dynamic modulus was calculated using Equation (17). For the numerical solution, a homogeneous finite element model was established (Figure 2a). The dynamic modulus curve obtained from the 2S2P1D parameters was converted into Prony series parameters for finite element input. Subsequently, dynamic simulation was performed using the SSD method to obtain the numerical dynamic modulus. Detailed simulation settings are provided in Section 3.2.

Figure 2b presents the master curves constructed from both the numerical and analytical solutions. It is evident that the simulated numerical curve aligns highly with the theoretical analytical curve (R^2^ = 0.9999). The Mean Absolute Percentage Error (MAPE) is as low as 2.37%, with a peak relative error across the full frequency domain of only 3.40%. This high degree of consistency fully validates the solver’s precision in handling complex stiffness matrices, thereby establishing a reliable computational framework for the subsequent mesoscale numerical simulation of heterogeneous materials.

## 3. Simulation Parameter Acquisition and Numerical Calculation

### 3.1. Acquisition of Interface Parameters

**Figure 3:**
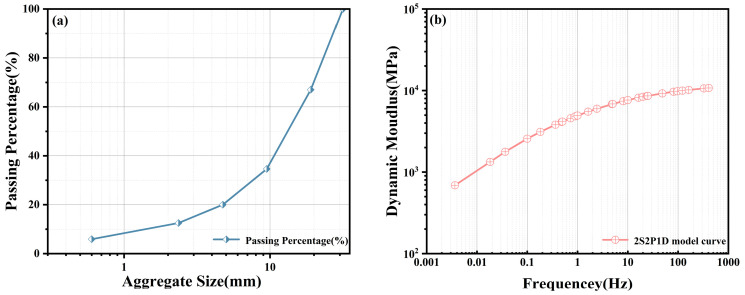
Material characteristics and rheological properties of the reference mixture. (**a**) Continuous aggregate gradation curve of the calcareous sand and (**b**) Master curve of ABCS.

Due to the microscopic dimensions of the ITZ, its dynamic viscoelastic parameters are difficult to obtain directly via mechanical testing. Consequently, interface parameters were derived via inverse analysis using micromechanical homogenization theory. This study utilized a 70# neat asphalt binder and a marine calcareous sand. It should be clarified that the term “calcareous sand” herein denotes the geological origin of marine biogenic sediments, which actually encompasses both coarse and fine aggregate fractions. Macroscopically, this material is characterized by high porosity and high crushability. Furthermore, this paper explicitly defines the ABCS formulated with the gradation curve shown in Figure 3a and an asphalt binder mass fraction of 14% as the reference mixture.

To prevent aggregate crushing during specimen fabrication, the materials were uniformly mixed at 165 °C, and cylindrical ABCS specimens (100 mm in diameter and 150 mm in height) were prepared using a customized Superpave Gyratory Compactor (SGC) protocol. The compaction effort was strictly limited to 15 gyrations under a maximum vertical pressure of 592 kPa.

Dynamic modulus tests were conducted using an IPC UTM-100 servo-hydraulic multifunctional material testing system (Moorabbin, Australia) to obtain the dynamic modulus at various temperatures. To ensure statistical reliability, three replicate specimens were tested for each condition. To determine the appropriate loading level, a strain amplitude sweep test was first conducted at each test temperature. Based on these preliminary results, a target strain amplitude of 100 µε was selected for the frequency sweep tests to ensure that the material response remained strictly within the Linear Viscoelastic (LVE) limit. The testing temperatures ranged from 0 °C to 30 °C (intentionally capped at 30 °C to avoid high-temperature aggregate crushing), with seven loading frequencies (0.1, 0.5, 1, 5, 10, 20, and 25 Hz). Subsequently, based on the TTSP, the dynamic modulus data obtained at different temperatures were shifted to construct a master curve. Specifically, the temperature shift factors ($a_{T}$) were determined simultaneously with the 2S2P1D model parameters (detailed in Section 2.1) by fitting the measured dynamic modulus data, with the shift factors following the WLF equation (Equation (15)). The resulting experimental master curve for ABCS at a reference temperature of T_0_ = 10 °C is presented in Figure 3b.

To extract ITZ parameters from the ABCS dynamic modulus, the three-phase spherical mesoscale model (Aggregate-ITZ-Asphalt Mastic Matrix) proposed by Christensen and Lo [74] was adopted. Based on the explicit solution for the effective modulus of isotropic composites derived by Weng [75,76], the complex modulus of the effective matrix can be expressed as follows:

(34) $$\frac{E_{eq}^{*}}{E_{m}^{*}}=1+\frac{\phi_{ITZ}(E_{ITZ}^{*}-E_{m}^{*})}{E_{m}^{*}+\beta_{0}(1-\phi_{ITZ})(E_{ITZ}^{*}-E_{m}^{*})}$$

(35) $$\beta_{0}=\frac{2(4-5\nu_{m})}{15(1-\nu_{m})}$$

where $E_{m}^{*}$ and $E_{ITZ}^{*}$ are the complex moduli of the asphalt mastic and the ITZ, respectively; $\phi_{ITZ}$ is the volume fraction of the ITZ; $\beta_{0}$ is a geometric parameter related to the Eshelby tensor; and $\nu_{m}$ is the Poisson’s ratio of the matrix.

The effective modulus of the overall asphalt mixture satisfies the following:

(36) $$\frac{E_{mix}^{*}}{E_{eq}^{*}}=1+\frac{\phi_{agg}(E_{agg}-E_{eq}^{*})}{E_{eq}^{*}+\beta_{0}(1-\phi_{agg})(E_{agg}-E_{eq}^{*})}$$

where $E_{agg}$ is the elastic modulus of the aggregate (treated as an elastic body), with an elastic modulus and Poisson’s ratio of 13,100 MPa and 0.2, respectively; and $\phi_{agg}$ is the aggregate volume fraction.

Based on the inverse analysis of the laboratory-measured macroscopic dynamic modulus master curve, the frequency-dependent dynamic modulus of the ITZ was obtained. The following empirical model was used to characterize the back-calculated ITZ enhancement factor:

(37) $$\frac{\left|E_{ITZ}^{*}(\omega )\right|}{\left|E_{m}^{*}(\omega )\right|}=A_{0}+\frac{A_{1}{\left(\omega /\omega_{ref}\right)}^{B_{1}}}{1+{\left(\omega /\omega_{c}\right)}^{B_{2}}}+D_{1}\log_{10}\left(\frac{\omega }{\omega_{ref}}\right)$$

where ω is the loading angular frequency; $\omega_{ref}$ is the reference angular frequency; $\omega_{c}$ is the characteristic angular frequency; and $A_{0}$, $A_{1}$, $B_{1}$, $B_{2}$, $D_{1}$ are fitting parameters. For the current loading condition, their values are 1.000, 0.985, −0.003, 0.387, and −0.111, respectively. This process ensures that the mesoscale parameters in the numerical model are energetically consistent with macroscopic experimental results and satisfy the Hashin–Shtrikman bounds [77] for composite mechanics. These values were converted into Prony series parameters, as detailed in Table 2.

### 3.2. Mesoscale Numerical Model of Asphalt-Bonded Calcareous Sand

The ABCS system is conceptualized as a three-phase composite consisting of the asphalt mastic matrix, calcareous sand aggregates, and the ITZ. Fine aggregates with particle sizes smaller than 2.36 mm were homogenized into the asphalt mastic matrix [78]. Utilizing the random aggregate generation algorithm described in Section 2.3, combined with the SSD simulation method, 50 sets of two-dimensional mesoscale models with dimensions of 100 mm × 150 mm were generated. The material parameters for the aggregates and ITZ are detailed in Section 3.1, while the viscoelastic parameters for the asphalt mastic are provided in Table 3.

**Figure 4:**
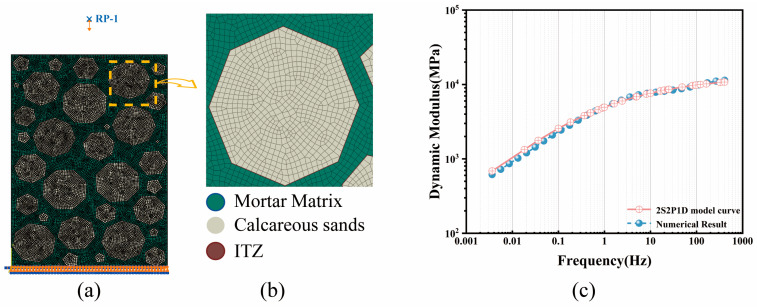
Mesoscale finite element model and simulation results: (**a**) Boundary conditions; (**b**) Local meshing of aggregates and ITZ; (**c**) Simulated master curve.

The boundary conditions were defined as follows: the bottom boundary of the model was set to “Encastre” (fully fixed), constraining all translational and rotational degrees of freedom. Regarding loading control, a displacement-controlled direct steady-state dynamic analysis was employed. To ensure the material response remained within the Linear Viscoelastic (LVE) range, a harmonic vertical displacement excitation corresponding to a strain amplitude of 100 µε was applied at the reference point, as illustrated in Figure 4a. A frequency sweep was conducted across a range from 0.004 Hz to 407.282 Hz, utilizing logarithmic sampling to compute the complex modulus response across the broadband frequency domain.

The numerically predicted master curve is compared with the experimental master curve constructed based on the 2S2P1D model in Figure 4c. The results demonstrate a high degree of agreement across the entire frequency spectrum (R^2^ = 0.99, MAPE = 6.94%). Both curves exhibit typical viscoelastic characteristics, with highly consistent asymptotic trends in the high-frequency glassy plateau and the low-frequency viscous flow region. This consistency confirms the capability of the proposed mesoscale mechanical model to independently reproduce macroscopic time–temperature superposition behaviors. Consequently, the model possesses the reliability required to predict mechanical responses under extreme conditions and is suitable for the subsequent in-depth analysis of mesoscale parameter sensitivity.

### 3.3. Independent Experimental Validation of the Mesoscale Model

To address the inherent localized circularity limitations of calibrating and validating a mesoscale model using a single dataset, a rigorous independent validation phase was introduced. A completely new ABCS mixture was designed and tested. This mixture maintained the identical aggregate gradation shown in Figure 3a, but the asphalt mass fraction was reduced to 12% (compared to the 14% baseline mixture used for calibration). Laboratory dynamic modulus tests were conducted on this modified formulation under identical temperature and frequency conditions to construct an independent experimental master curve.

Subsequently, 10 independent 2D RVE models with dimensions of 100 mm × 150 mm (matching the experimental specimen size) were generated using the random aggregate algorithm to ensure statistical reliability and eliminate random microstructural bias. Crucially, to independently verify the physical transferability of the interfacial properties, the viscoelastic parameters of the ITZ back-calculated in Section 3.1 were strictly kept unchanged. The only variables adjusted in these numerical models were the spatial distribution and the reduced volume fraction of the asphalt mastic matrix. The SSD solver was then employed to predict the broadband dynamic response of this new system, and the average dynamic modulus from the 10 models was utilized.

**Figure 5:**
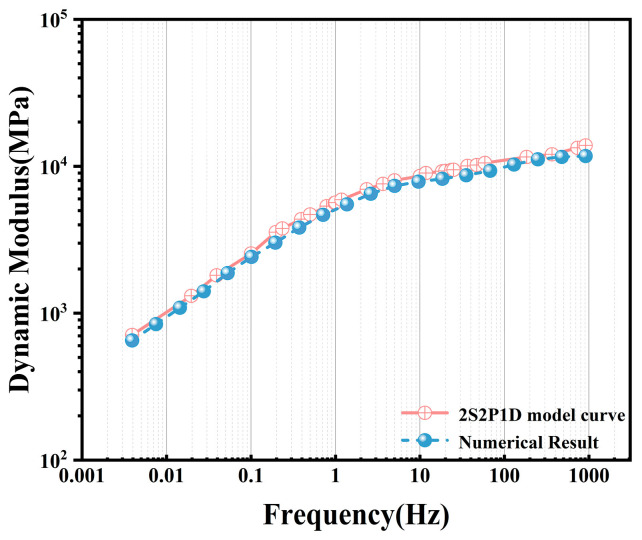
Independent validation: Comparison of experimental and average SSD-predicted master curves for the ABCS mixture with a 12% asphalt mass fraction.

Figure 5 presents the comparison between the average SSD-predicted master curve and the newly acquired experimental master curve for the validation mixture. The numerical predictions exhibit excellent agreement with the independent experimental data across the entire frequency spectrum (R^2^ = 0.96, MAPE = 9.73%). This high degree of consistency effectively eliminates the circularity concern. Furthermore, it confirms that the theoretically derived ITZ parameters are not merely mathematical artifacts of the baseline calibration, but possess robust physical validity and predictive reliability across different mixture designs.

To further decouple and quantify the influence of key mesostructural and compositional parameters—specifically the maximum aggregate size, ITZ thickness, asphalt binder content, and the RVE size—a comprehensive large-scale simulation matrix was established. This study constructed models across varying parameter ranges, comprising five groups of maximum aggregate sizes (23.5 mm, 25.5 mm, 27.5 mm, 29.5 mm, and 31.5 mm), five variations of ITZ thickness (0 µm, 25 µm, 50 µm, 75 µm, and 100 µm), two variations of asphalt mass fraction (14% baseline and 12% reduced content), and nine distinct RVE sizes (100 mm, 125 mm, 150 mm, 175 mm, 200 mm, 225 mm, 250 mm, 275 mm, and 300 mm). In total, 860 independent mesoscale models were generated and subjected to steady-state dynamic simulation to facilitate a robust sensitivity analysis.

## 4. Results and Discussion

Based on the comprehensive validation of the model’s broadband prediction capabilities in Section 3, this section further leverages the predictive advantages of the proposed mesoscale numerical model to conduct an in-depth parametric sensitivity analysis. Unlike traditional laboratory testing, which is often constrained by equipment frequency limits and sample variability, numerical simulation enables precise control over single variables and elucidates material behavior across a broader frequency domain.

Given the significant time–temperature dependence of ABCS, the mechanisms governing its mechanical response vary fundamentally across different loading states. Governed by the TTSP, the analyzed reduced frequency range (10^−3^ Hz to 10^3^ Hz) is essential for fully capturing this broadband viscoelastic behavior [79,80]. Analyzing this shifted domain holds significant practical relevance: extremely low reduced frequencies (10^−3^ Hz) correspond to high-temperature or slow-moving traffic conditions, which is critical for evaluating rutting susceptibility. Conversely, high reduced frequencies (10^3^ Hz) represent low-temperature or high-speed traffic conditions, essential for assessing cracking resistance.

Within this framework, this section systematically investigates the influence of maximum aggregate size, ITZ thickness, asphalt binder content, and RVE size on the macroscopic dynamic modulus. The objective is to reveal the cross-scale mapping mechanisms linking mesostructural and compositional parameters to macroscopic mechanical performance.

### 4.1. Analysis of the Influence of Maximum Aggregate Size on Dynamic Modulus

To investigate the contribution of the maximum aggregate size ($d_{\max}$) within the gradation to the global dynamic modulus of ABCS, five groups of random aggregate models were established with varying maximum aggregate sizes (23.5 mm, 25.5 mm, 27.5 mm, 29.5 mm, and 31.5 mm), while maintaining all other gradation characteristics constant. To eliminate the interference of boundary effects on computational accuracy, the dimensions of all simulation models were standardized to 300 mm × 300 mm. On this basis, dynamic modulus calculations and comparative analyses were conducted for each group across the full frequency domain.

**Figure 6:**
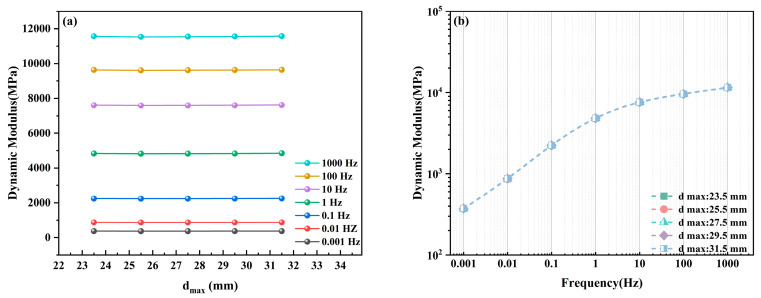
Numerical simulation results for different maximum aggregate sizes: (**a**) Dynamic modulus at discrete frequencies; (**b**) Comparison of master curves.

Figure 6 illustrates the trend of dynamic modulus variation with respect to maximum aggregate size under different loading frequencies. It is evident that the dynamic modulus curves at each frequency manifest as approximately horizontal lines. This indicates that within the selected range of particle size variation (23.5 mm–31.5 mm), alterations in the maximum aggregate size do not induce significant fluctuations in the dynamic modulus. Figure 6b further presents a comparison of the dynamic modulus master curves for the different maximum aggregate sizes; the five curves exhibit near-perfect overlap with no observable dispersion.

These results indicate that, within the current two-dimensional mesoscale numerical simulation framework, the dynamic modulus of ABCS is insensitive to variations in maximum aggregate size. The underlying physical mechanism may be attributed to two primary factors. First, according to composite mechanics theory, the volume fraction of each constituent is the primary determinant of the equivalent modulus. Since all model groups maintained a constant total aggregate volume fraction, minor adjustments to the maximum aggregate size did not alter the total proportion of aggregates within the RVE; consequently, the macroscopic stiffness baseline remained stable. Second, although larger aggregates theoretically provide stronger skeletal interlocking, the viscoelastic behavior of the asphalt matrix in this bonded system largely dominates the global response, effectively masking the skeletal reinforcement effects resulting from minor variations in aggregate size. Therefore, within the range of standard engineering applications, adjustments to the maximum particle size have a negligible impact on the macroscopic viscoelastic stiffness of the material.

### 4.2. Analysis of the Influence of ITZ Thickness on Dynamic Modulus

The ITZ serves as the microscopic boundary region between aggregate particles and the asphalt mastic matrix. Its physicochemical properties and mechanical behaviors typically differ significantly from those of the bulk asphalt mastic or the aggregates. This is particularly pronounced in calcareous sand, where significant intra-granular porosity and high angularity trigger complex physicochemical interactions at the interface, thereby altering local rheological properties. To quantify the impact of these mesoscopic mechanisms on macroscopic viscoelasticity, this study utilized a 300 mm × 300 mm RVE model to systematically simulate five gradient levels of ITZ thickness: 0 µm, 25 µm, 50 µm, 75 µm, and 100 µm. To eliminate stochastic errors arising from random aggregate distribution and to ensure statistical significance, 50 independent mesoscale models were randomly generated and analyzed for each thickness condition.

**Figure 7:**
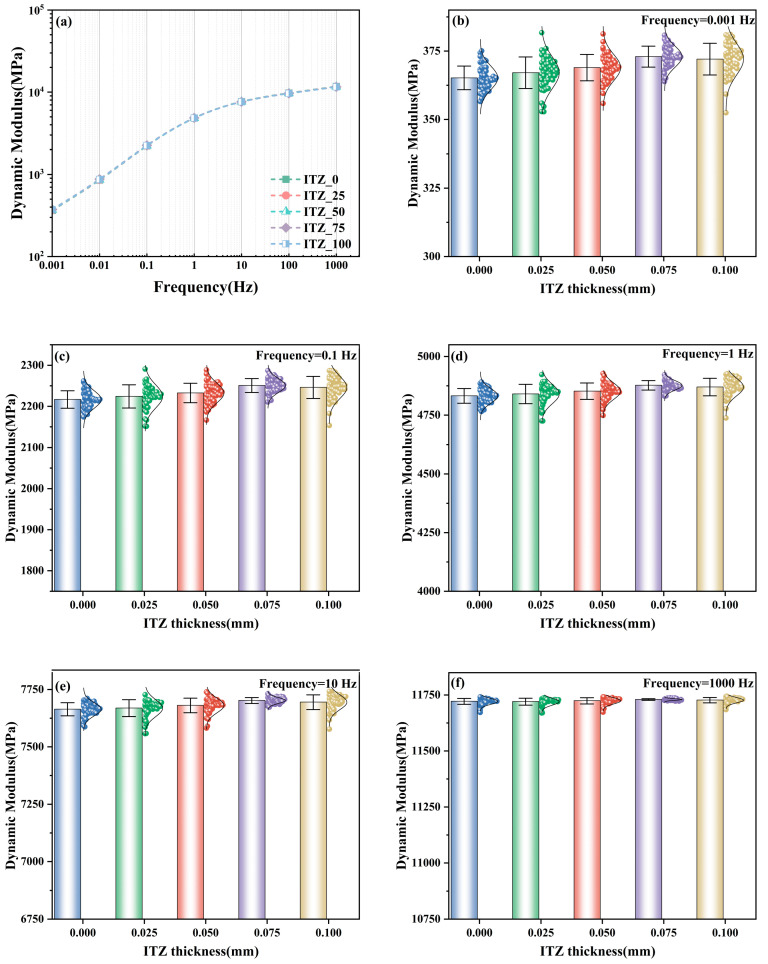
Comparison of dynamic modulus master curves under varying ITZ thicknesses. (**a**) Comparison of dynamic modulus of different interface layer thickness; (**b**) Frequency 0.001 Hz dynamic modulus figure; (**c**) Frequency 0.1 Hz dynamic modulus figure; (**d**) Frequency 1 Hz dynamic modulus figure; (**e**) Frequency 10 Hz dynamic modulus figure; (**f**) Frequency 1000 Hz dynamic modulus figure.

Figure 7a presents the dynamic modulus master curves of ABCS across a broadband frequency range under varying ITZ thicknesses. The results demonstrate that, for all conditions, the dynamic modulus exhibits a typical monotonic increase with loading frequency. Notably, the master curves corresponding to different ITZ thicknesses virtually overlap without distinct separation. This suggests that the macroscopic viscoelasticity of ABCS is intrinsically governed by the rheological properties of the bulk binder and the loading frequency. The variation in ITZ thickness does not alter the fundamental shape of the master curve, indicating that the interfacial layer acts as a mesoscopic perturbation rather than a dominant factor in the global viscoelastic model.

To further elucidate the frequency-dependent influence of the ITZ, Figure 7b–f detail the statistical distribution of the dynamic modulus at five characteristic frequencies (0.001 Hz, 0.1 Hz, 1 Hz, 10 Hz, and 1000 Hz). In these figures, the bar charts, scatter plots, and error bars represent the mean, data dispersion, and standard deviation, respectively.

At low frequencies (0.001–1 Hz), corresponding to high-temperature service or long-term loading, the dynamic modulus exhibits a non-monotonic trend with increasing ITZ thickness, indicating a threshold effect. As the ITZ thickness increases from 0 µm to 75 µm, the mean dynamic modulus shows a slight increase. While microscopic physicochemical interactions and mineralogy are not geometrically discrete in this mesoscale continuum model, their macroscopic effects—such as binder absorption and interface stiffening—are mathematically homogenized into the ITZ viscoelastic parameters. Specifically, the highly developed intra-particle porosity and strong physical adsorption capacity of calcareous sand promote a high-stiffness interfacial network, enhancing stress transfer when the bulk matrix softens. However, when the thickness is further increased to 100 µm, the mean modulus exhibits a slight decline. This implies a diminishing marginal return: an excessively thick ITZ results in significant volumetric consumption of the asphalt mastic, reducing the volume of effective bulk binder available for cohesion. Furthermore, excessive overlap of interfacial zones between adjacent particles may compromise the continuity of the matrix, thereby offsetting part of the strengthening effect. Conversely, under high-frequency loading conditions (10 Hz–1000 Hz), the influence of ITZ thickness diminishes progressively. At 1000 Hz, the statistical differences in dynamic modulus values among the groups are negligible, indicating that the material becomes insensitive to interfacial heterogeneity at high frequencies.

In summary, an optimal ITZ thickness threshold exists (approximately 75 µm) that maximizes deformation resistance at low frequencies (high temperatures) without compromising performance at high frequencies (low temperatures). This suggests that optimizing mixing protocols to induce moderate interfacial interaction is an effective strategy for balancing the high-temperature rutting resistance and low-temperature cracking resistance of pavements in reef and island environments.

### 4.3. Analysis of the Influence of Asphalt Binder Content on Dynamic Modulus

To address the practical engineering implications of mixture design and to evaluate the model’s parametric sensitivity to macro-level composition, the influence of asphalt binder content on the dynamic modulus was investigated. In addition to the baseline 14% asphalt mass fraction mixture, a comparative mesoscale model group with a reduced asphalt content of 12% was established. To eliminate boundary effect interference and maintain consistency with previous parametric analyses, the dimensions of the simulation models in this section were standardized to large-scale 300 mm × 300 mm configurations, with the ITZ thickness maintained at 75 µm.

For the newly introduced 12% asphalt content group, 10 independent random aggregate models were generated. It is worth noting that, as demonstrated in the subsequent RVE size analysis (detailed in Section 4.4) using the baseline mixture, a 300 mm dimension sufficiently exceeds the statistical homogeneity threshold, resulting in minimal macroscopic variance. Therefore, a sample size of 10 for these large-scale RVEs provides robust statistical reliability for extracting the mean dynamic modulus while significantly optimizing computational efficiency.

**Figure 8:**
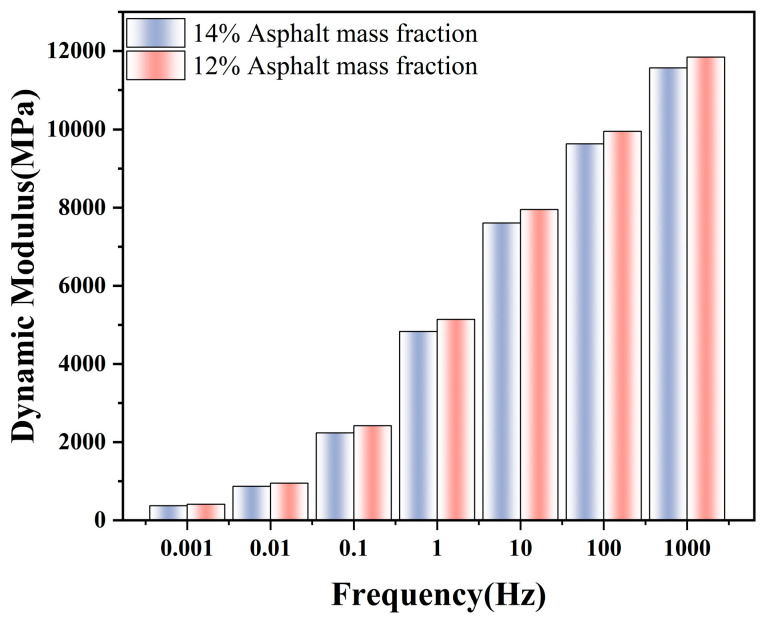
Influence of asphalt binder content on the macroscopic dynamic modulus of ABCS.

Figure 8 illustrates the average dynamic modulus master curves comparing the 12% and 14% asphalt mass fractions. The results indicate a distinct negative correlation between asphalt content and macroscopic stiffness. Reducing the asphalt mass fraction from 14% to 12% visibly increases the global dynamic modulus across the entire frequency spectrum, with the enhancement being particularly pronounced in the low-frequency (high-temperature) domain. Physically, a lower binder content reduces the thickness of the effective viscoelastic mastic film between aggregates. This reduction enhances the direct skeletal interlocking and contact friction among the rigid calcareous sand particles. Conversely, the 14% reference mixture physically pushes the aggregates slightly further apart, allowing the softer, temperature-susceptible asphalt matrix to dominate the global viscoelastic response. This numerical trend confirms that reasonably reducing the binder content improves high-temperature rutting resistance, aligning with classical asphalt mixture mechanics and proving the framework’s capability to virtually optimize the sand-binder ratio.

### 4.4. Analysis of the Influence of RVE Size on Dynamic Modulus

ABCS exhibits significant mesostructural heterogeneity due to the randomness of aggregate size, morphology, and spatial distribution. This internal non-uniformity is the fundamental cause of the size effect in composite materials [81]. Therefore, conducting a systematic sensitivity analysis of the RVE size effect is crucial for ensuring the reliability and precision of numerical simulation results. The objective of this section is to determine the rational RVE size for the ABCS composite to effectively eliminate the size effect, thereby obtaining macroscopic mechanical properties that are independent of model dimensions.

To isolate the influence of RVE size from other mesoscopic features, the aggregate content, gradation, and interface layer thickness were kept constant. The statistical distribution characteristics of the dynamic modulus were investigated under varying loading frequencies using models of different sizes. This study established nine distinct RVE sizes: 100 mm, 125 mm, 150 mm, 175 mm, 200 mm, 225 mm, 250 mm, 275 mm, and 300 mm. For each RVE size, 50 model samples with different random microstructures were generated for statistical analysis.

**Figure 9:**
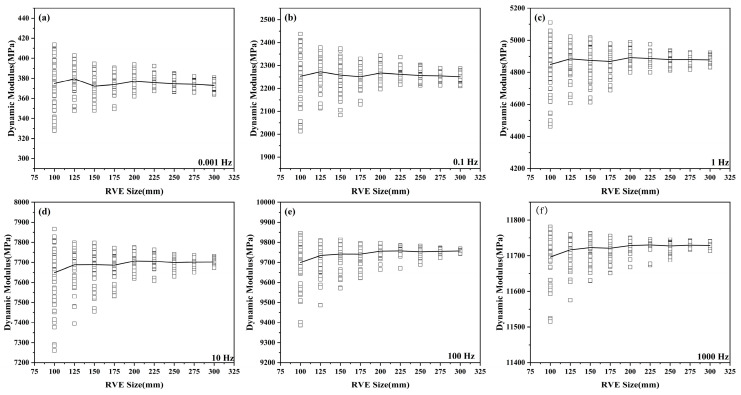
Statistical distribution of dynamic modulus under different loading frequencies: (**a**) 0.001 Hz; (**b**) 0.1 Hz; (**c**) 1 Hz; (**d**) 10 Hz; (**e**) 100 Hz; (**f**) 1000 Hz.

Figure 9 illustrates the scatter distribution of the dynamic modulus as a function of model size under different loading frequencies. It is observed that across all frequency ranges, the dispersion of the dynamic modulus gradually decreases as the RVE size increases. When the RVE size reaches 250–300 mm, the mean dynamic modulus stabilizes, and the standard deviation becomes minimal. This indicates that above this threshold, microstructural distribution and boundary effects are negligible. When the RVE size is small, the local heterogeneity introduced by the random distribution of aggregates dominates, causing significant stiffness discrepancies between different models and making them susceptible to local boundary effects, thereby inducing large fluctuations in the dynamic modulus. As the RVE size increases, the local effects of random distribution and orientation are progressively averaged out, allowing microstructural characteristics to be more uniformly reflected in the global response. Beyond the critical size, further increasing the RVE does not affect the system’s macroscopic modulus, indicating that the RVE has achieved statistical representativeness.

### 4.5. Analytical Determination of RVE Size

The analysis above demonstrates that simulation error varies significantly with RVE size. To further determine a reasonable RVE model size, a statistical approach was employed to analyze the dispersion of the dynamic modulus predicted by models of different sizes. An exponential function was adopted to describe the relationship between the physical properties of the material and its size [82,83]:

(38) $$D_{c}^{2}(A)=D_{c}^{2}\times {\left(\frac{S}{A}\right)}^{\alpha }$$

where $D_{c}^{2}(A)$ is the variance of the sample when the specimen size is A; $D_{c}^{2}$ is the point variance of the random variable; S is a geometric parameter; and α is the power exponent.

Equation (38) can be linearized logarithmically as follows:

(39) $$\log\left[D_{c}^{2}\left(A\right)\right]=-\alpha \log\left(A\right)+\left[\log\left(D_{c}^{2}\right)+\alpha \log\left(S\right)\right]$$

For a two-phase material with moduli $D_{1}^{*}$ (Phase 1) and $D_{2}^{*}$ (Phase 2), the point variance $D_{c}^{2}$ is given by the following [83]:

(40) $$D_{c}^{2}=f(1-f){\left(D_{1}^{*}-D_{2}^{*}\right)}^{2}$$

Extending Equation (40) to multiphase materials yields the following:

(41) $$D_{c}^{2}=\sum_{i=1}^{3}{\left(D_{i}^{*}\right)}^{2}f_{i}-{\left(\sum_{i=1}^{3}D_{i}^{*}f_{i}\right)}^{2}$$

where $f_{i}$ is the volume fraction of each constituent phase of the Asphalt-Bonded Calcareous Sand, and $D_{i}^{*}$ is the modulus of each phase.

**Figure 10:**
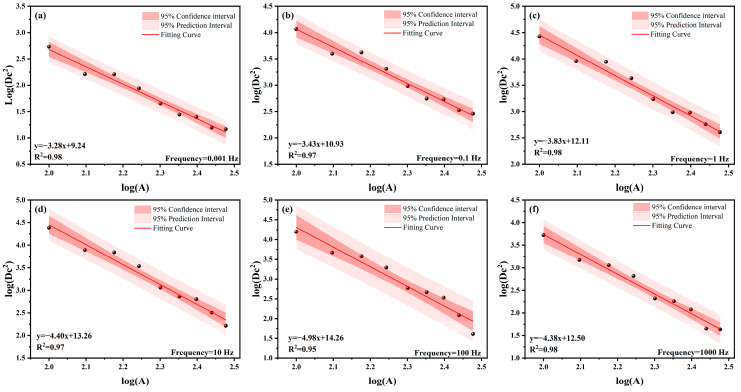
Fitting results of dynamic modulus dispersion for different RVE sizes across various frequencies. (**a**) 0.01 Hz fitting results; (**b**) 0.1Hz fitting results; (**c**) 1 Hz fitting results; (**d**) 10 Hz fitting results; (**e**) 100 Hz fitting results; (**f**) 1000 Hz fitting results.

Substituting the numerical simulation results into Equation (39) allows for the solution of parameters S and α. The fitting results are presented in Figure 10 and Table 4. As shown in the figure, $\log\left(D_{c}^{2}\right)$ decreases as $\log\left(A\right)$ increases, indicating a strict linear declining trend in data dispersion with increasing model size across the full frequency domain. Furthermore, the coefficient of determination (R^2^) exceeds 0.95 for all cases, demonstrating that this function well characterizes the statistical distribution laws of the random aggregate model’s dynamic modulus. Further analysis reveals that the absolute value of the fitting slope exhibits distinct frequency-domain evolution characteristics: the value climbs from 3.28 at 0.001 Hz to 4.98 at 100 Hz, reflecting the accelerating effect of matrix stiffening on statistical homogenization at high frequencies. However, the slope regresses to 4.38 at 1000 Hz, suggesting that the convergence rate stabilizes after the material enters the glassy state. Based on this, the large modulus contrast under low-frequency (high-temperature) conditions leads to convergence hysteresis; therefore, this condition serves as the controlling case for determining RVE size.

According to mathematical statistics theory [83], the size of the calculated RVE is correlated with the desired precision ε:

(42) $$\varepsilon =\frac{2D_{c}(A)}{M_{0}\sqrt{N_{0}}}$$

where $M_{0}$ is the mean value, and $N_{0}$ is the number of independent samples. Substituting Equation (42) into Equation (38), the RVE size A for a given expected error ε can be expressed as follows:

(43) $$A=S{\left(\frac{4D_{c}^{2}}{\varepsilon^{2}M_{0}^{2}N_{0}}\right)}^{1/\alpha }$$

In this context, the fault-tolerance criterion for the RVE is quantitatively defined as the allowable relative error, ε, in the predicted dynamic modulus. A smaller ε signifies a stricter tolerance, necessitating a larger RVE size to achieve the desired accuracy.

**Figure 11:**
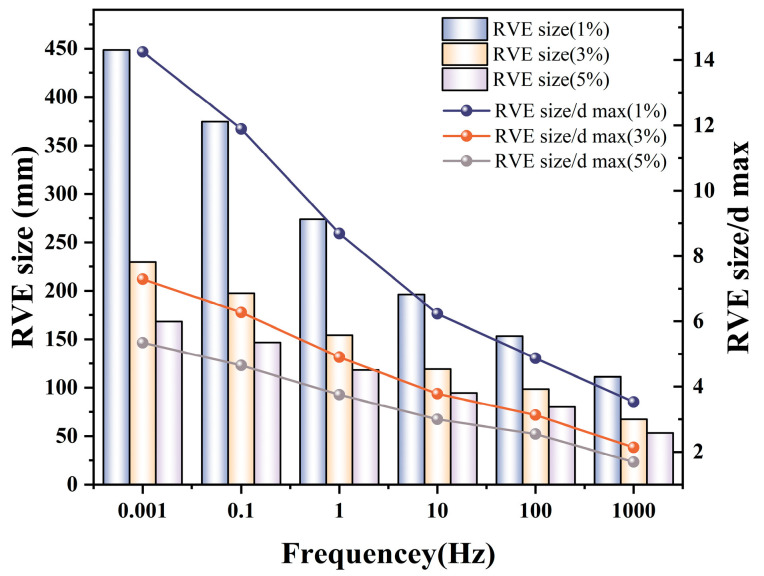
Estimated appropriate RVE sizes under different frequencies and error tolerances.

By substituting the fitted data from Table 4 into Equation (43), the estimated appropriate RVE sizes for different frequencies and expected errors are obtained, as shown in Figure 11. We systematically analyzed three representative values of ε: 1%, 3%, and 5%. The 1% criterion represents a high-precision requirement for in-depth mechanistic studies, while the 5% criterion reflects a practical tolerance for routine engineering applications. When the expected error is 1%, the required RVE size ranges from approximately 111 to 449 mm, corresponding to 3.5–14.3 times the maximum aggregate size ($d_{\max}$). For an expected error of 3%, the size ranges from 67 to 230 mm (2.1–7.3 times $d_{\max}$). When the expected error is relaxed to 5%, the RVE size ranges from 53 to 168 mm, which is 1.7–5.3 times $d_{\max}$. The results demonstrate a significantly low-frequency sensitivity in RVE size requirements. At low frequencies (corresponding to high temperatures), the asphalt matrix is extremely soft while the aggregates remain rigid, maximizing the modulus contrast between the two phases. This extreme stiffness mismatch causes severe fluctuations in the local stress field, necessitating a larger volume to smooth out this heterogeneity. As the frequency increases (corresponding to low temperatures), the asphalt matrix transitions into a glassy state, significantly increasing its modulus and reducing the stiffness differential with the aggregates. The material tends to become mechanically more homogeneous, thus allowing smaller dimensions to satisfy the same error requirements.

These observations align with reported mechanics trends. Kim et al. [84] confirm macroscopic responses exhibit insensitivity to maximum aggregate size. Wei et al. [42] prove interfacial networks dominate load transfer during high-temperature matrix softening. Crucially, our RVE criterion (1.7–5.3 times $d_{\max}$) bridges theoretical mechanics and pavement engineering. It obeys statistical convergence principles by Kanit et al. [82] and aligns with asphalt mixture mechanics trends from Cao et al. [61], requiring comparable multiples of maximum aggregate size.

### 4.6. Applicability and Limitations of 2D Simplification

It is worth noting that while actual calcareous sand aggregates possess complex three-dimensional (3D) spatial morphologies, this study adopted a 2D mesoscale model as the foundational framework for executing large-scale parametric sensitivity analyses. This strategic choice was governed by the critical need for computational efficiency in high-throughput modeling. Constructing and solving high-fidelity 3D mesoscale models with realistic, irregular aggregate geometries remains a formidable challenge due to the immense computational costs and the complexity of 3D mesh generation. For example, existing literature indicates that applying the SSD method to a simplified 3D spherical model (3 mm in side length) for predicting dynamic moduli at merely six frequencies (from 10^−2^ to 10^3^ Hz) requires 83 min [62]. In stark contrast, utilizing the proposed 2D SSD framework, completing the broadband SSD calculations (10^−3^ Hz to 10^3^ Hz) for a batch of 10 massive 300 mm RVEs requires approximately only 6.5 min per model on a standard workstation. Given that this study involves an unprecedented 920 independent mesoscale models to capture the multi-scenario influences of particle size, ITZ thickness, and RVE dimensions, a 3D approach would be computationally intractable. Furthermore, previous studies [61,85] have demonstrated that 2D models robustly capture the frequency-dependent viscoelastic trends of asphalt mixtures.

Despite these advantages, the inherent mechanical limitations of 2D simplification must be objectively acknowledged. First, the macroscopic strength of asphalt mixtures heavily relies on the physical interlocking and friction among aggregate particles in 3D space [86]. By reducing the system to two dimensions, the number of effective contact points between particles is significantly decreased, and out-of-plane confinement effects are lost. Consequently, 2D models may underestimate the true skeletal stability and the structural reinforcement provided by the 3D aggregate packing [86,87].

Second, the spatial configuration of the Interfacial Transition Zone (ITZ) and the resulting load transfer pathways are fundamentally altered in 2D. In realistic 3D systems, ITZs form a “conjunctive shell” mechanism that wraps around aggregates, creating a continuous reinforcing network [88]. In a 3D environment, micro-stress chains can propagate through diverse spatial pathways, effectively bypassing localized weak zones or voids. Conversely, 2D models restrict stress transfer pathways to a single plane. This constraint reduces the efficiency of load distribution and may artificially amplify localized stress concentrations, altering the topological characteristics of the stress network compared to the actual material [42].

To bridge the gap between 2D efficiency and 3D fidelity, future research will focus on reconstructing realistic 3D RVE models using non-destructive X-ray Computed Tomography (X-CT). To overcome the aforementioned computational bottlenecks of 3D modeling, the integration of Artificial Intelligence (AI) and machine learning-driven surrogate models presents a promising frontier. By training deep learning networks on large-scale simulation datasets, it will be possible to predict the complex 3D mechanical response of heterogeneous ABCS with high precision and significantly reduced temporal costs, thereby refining the engineering applicability of the framework.

## 5. Conclusions

This study established a mesoscale numerical simulation framework for Asphalt-Bonded Calcareous Sand (ABCS) based on a random particle generation algorithm and direct Steady-State Dynamics (SSD) analysis. By generating and calculating 920 sets of two-dimensional mesoscale models, the validity of this method for broadband prediction was verified, and the cross-scale mapping mechanisms of maximum aggregate size, Interfacial Transition Zone (ITZ) thickness, and RVE size on the macroscopic broadband viscoelastic response were systematically revealed. The main conclusions are as follows:

By bridging explicitly mapped mesoscale heterogeneities directly with a frequency-domain SSD solver, this approach circumvents the severe computational bottlenecks of time-domain step-by-step integration. Validation results demonstrate that the model independently reproduces broadband master curves conforming to the Time–Temperature Superposition Principle (TTSP) with extremely high precision (baseline R^2^ = 0.99, MAPE = 6.94%; independent 12% validation R^2^ = 0.96, MAPE = 9.73%). Furthermore, the framework quantifiably accelerates the simulation process: completing broadband SSD calculations (10^−3^ Hz to 10^3^ Hz) for a batch of 10 massive 300 mm RVEs requires only approximately 6.5 min in total. This offers a radical efficiency advantage over conventional time-domain FEM, which requires an intractable number of increments to span six orders of frequency magnitude.

In addition to these methodological advancements, the systematic parametric analysis revealed that the macroscopic responses of ABCS exhibit insensitivity to maximum aggregate size but distinct sensitivity to interfacial characteristics and binder content. Specifically, reducing the asphalt mass fraction (e.g., from 14% to 12%) significantly enhances the low-frequency dynamic modulus by promoting the direct skeletal interlocking of calcareous sand particles. An optimal ITZ thickness threshold of approximately 75 µm was also identified, which maximizes low-frequency deformation resistance. To ensure statistical reliability in such simulations, the convergence criterion for the RVE was analytically determined: to satisfy a practical engineering error tolerance of 5%, an RVE side length of 1.7–5.3 times the maximum aggregate size is required.

From a practical engineering perspective, these established RVE criteria provide pavement designers with quantitative geometric boundaries for “virtual mix design,” ensuring statistical reliability while preventing computational waste. Furthermore, the findings provide direct mixture design guidance: reasonably reducing the asphalt binder content to enhance skeletal interlocking, alongside optimizing mixing protocols to achieve a moderate ~75 µm ITZ, can effectively balance high-temperature rutting resistance and low-temperature cracking resistance in island and reef pavement engineering.

Despite the balance achieved between computational efficiency and physical elucidation, certain limitations remain to be addressed in future research. First, 2D planar models inherently lack out-of-plane degrees of freedom, which may partially underestimate spatial skeletal interlocking. Future work will reconstruct realistic 3D RVE models using X-ray computed tomography (X-CT) to explicitly quantify the complex 3D impact of intra-particle porosity and localized binder absorption—features that were simplified as homogenized properties in the current study. Second, while this study evaluated the influence of binder content, more definitive conclusions require broader experimental campaigns involving systematic aggregate content variations. Subsequent research phases will also introduce Nano-indentation and Atomic Force Microscopy (AFM) to conduct in situ mechanical testing of the ITZ, further refining the engineering applicability of the ABCS framework.

## Figures and Tables

**Table 1:** Assumed parameter values for the validation model.

$E_{0}$	$E_{g}$	k	h	α	β	τ
500	20,000	0.4	0.02	2	10	0.02

**Table 2:** Viscoelastic parameters of the Interfacial Transition Zone (ITZ).

*i*	1	2	3	4	5	6	7	8	9	10	11	12	$E_{e}$
$\tau_{i}$	8.02 × 10^−4^	4.85 × 10^−3^	2.93 × 10^−2^	1.77 × 10^−1^	1.07	6.47	3.91 × 10^1^	2.37 × 10^2^	1.43 × 10^3^	8.64 × 10^3^	5.23 × 10^4^	3.16 × 10^5^	107.37 MPa
$g_{i}$	3.61 × 10^−1^	1.00 × 10^−13^	2.47 × 10^−1^	1.73 × 10^−1^	1.12 × 10^−1^	5.23 × 10^−2^	2.45 × 10^−2^	1.11 × 10^−2^	3.13 × 10^−3^	2.04 × 10^−3^	1.00 × 10^−13^	2.37 × 10^−4^	

**Table 3:** Viscoelastic parameters of the asphalt mastic.

*i*	1	2	3	4	5	6	7	8	9	10	11	$E_{e}$
$\tau_{i}$	9.31 × 10^−4^	6.54 × 10^−3^	4.59 × 10^−2^	3.23 × 10^−1^	2.27	1.59 × 10^1^	1.12 × 10^2^	7.85 × 10^2^	5.51 × 10^3^	3.87 × 10^4^	2.72 × 10^5^	39.95 MPa
$g_{i}$	4.88 × 10^−1^	7.72 × 10^−2^	2.58 × 10^−1^	1.05 × 10^−1^	4.35 × 10^−2^	1.44 × 10^−2^	6.89 × 10^−3^	1.61 × 10^−3^	1.42 × 10^−3^	6.33 × 10^−5^	1.00 × 10^−13^	

**Table 4:** Fitted parameter values.

Frequency (Hz)	$D_{c}^{2}$	S	α
0.001	41,097,361.27	3.13	3.28
0.1	37,055,384.77	9.55	3.43
1	29,592,725.06	16.26	3.83
10	19,073,856.09	22.86	4.40
100	13,867,058.89	26.87	4.98
1000	5,600,440.22	20.57	4.38

## Data Availability

The original contributions presented in this study are included in the article. Further inquiries can be directed to the corresponding authors.

## Abbreviations

The following abbreviations are used in this manuscript:

ABCS	Asphalt-Bonded Calcareous Sand
SSD	Steady-State Dynamics
ITZ	Interfacial Transition Zone
RVE	Representative Volume Element
TTSP	Time–Temperature Superposition Principle

## Nomenclature

H(t)	Heaviside step function	$P^{N}$	External load vector
ε(t)	Strain function	$D^{el}$	Elasticity matrix
$\Delta \varepsilon_{i}$	Strain increment applied at time $\tau_{i}$	$K^{NM}$	Stiffness matrix
σ(t)	Stress response	$C_{\left(k\right)}^{NM}$	Stiffness proportional viscous damping matrix
$E_{R}(t)$	Stress relaxation modulus	$C_{\left(s\right)}^{NM}$	Stiffness proportional structural damping matrix
$E_{e}$	Equilibrium modulus	$ℜ\left(u^{M}\right)$	Real parts of the amplitudes of the displacement
$E^{\prime }(\omega )$	Storage modulus	$ℑ\left(u^{M}\right)$	Imaginary parts of the amplitudes of the displacement
$E^{\prime \prime }(\omega )$	Loss modulus	$ℜ\left(P^{N}\right)$	Real parts of the amplitude of the force applied to the structure
$E^{*}$	Complex modulus	$ℑ\left(P^{N}\right)$	Imaginary parts of the amplitude of the force applied to the structure
$\left|E^{*}\right|$	Dynamic modulus	$E_{0}$	Static modulus
δ	Phase angle	$E_{g}$	Glassy modulus
$a_{T}$	Time–temperature shift factor	$D_{c}^{2}(A)$	Sample variance
$C_{1}$	Equation parameters	$D_{c}^{2}$	Unknown parameter
$C_{2}$	Equation parameters	S	Geometric parameters
$M^{NM}$	Mass matrix	α	Power index
$C_{\left(m\right)}^{NM}$	Mass proportional damping matrix	$f_{i}$	Volume fraction of each phase
$I^{N}$	Internal load vector	$D_{i}^{*}$	Modulus of each phase
